# Biobased Films Based on Chitosan and Microcrystalline Cellulose for Sustainable Packaging Applications

**DOI:** 10.3390/polym16050568

**Published:** 2024-02-20

**Authors:** Erika Alessia Di Liberto, Nadka Tzankova Dintcheva

**Affiliations:** 1 Dipartimento di Ingegneria, Università di Palermo, Viale delle Scienze, Ed. 6, 90128 Palermo, Italy; 2 INSTM-National Interuniversity Consortium of Materials Science and Technology-Board of Sustainability of INSTM, Via G. Giusti, 9, 50121 Firenze, Italy

**Keywords:** sustainable composites, microcrystalline cellulose, chitosan, biodegradable materials, packaging films

## Abstract

The transition to a more sustainable lifestyle requires a move away from petroleum-based sources and the investigation and funding of renewable and waste feedstocks to provide biobased sustainable materials. The formulation of films based on chitosan and microcrystalline cellulose with potential applications in the packaging sector has been demonstrated. Glycerol is also used as a plasticizer in the formulation of flexible films, while mucic acid is used as a valid alternative to acetic acid in such films. The film based on chitosan, microcrystalline cellulose, glycerol, and mucic acid shows properties and a performance similar to those of the film formulated with acetic acid, and, in addition, it seems that the photo-oxidation resistance of the film based on mucic acid is better than that of the material containing acetic acid. The films were characterized using spectroscopy (FTIR and UV-vis), tensile testing, water contact angle measurements, surface observations, and photo-oxidation resistance measurements. The presence of microcrystalline cellulose enhances the mechanical behavior, UV barrier properties, and surface hydrophobicity of the film. The feasibility of formulating chitosan-based films, with or without microcrystalline cellulose, which exhibit good properties and performances is demonstrated. Mucic acid instead of acetic acid is used in the formulation of these film.

## 1. Introduction

The vast increase in solid waste and climate change can be attributed to an overdependence on fossil fuels and global industrialization. Currently, primary global challenges include combating resource depletion and pollution, reducing greenhouse gas emissions, and optimizing waste management. Countries worldwide have made efforts to mitigate climate change by setting emission reduction standards or by using advanced technologies to limit greenhouse gas emissions [1,2]. The European Commission has adopted several strategic initiatives to promote a circular economy and sustainable development in Europe, concepts that are closely intertwined and key to addressing environmental and economic challenges; these recommendations include ideas from different European committees [3,4,5].

A circular economy requires the production of goods in a more sustainable way, using renewable energy sources, minimizing waste, reusing materials in multiple production cycles, and recycling resources that cannot be reused. This extends the life cycle of products and helps to minimize the generation of waste. In fact, once the product has fulfilled its function, the materials from which it is made should be recycled wherever possible, creating additional value, thus facilitating the movement from a linear to a circular economy [6]. 

Sustainable composites made from renewable materials, waste, and biopolymer matrices are of particular interest as they have the potential to reduce environmental impacts and support an efficient circular economy [7]. Several biopolymers, such as polylactic acid (PLA) [8], starch-based polymers [9], or polyhydroxyalkanoates [10], have been proposed as suitable materials for biocomposites’ preparation. Furthermore, biocomposites made from biopolymers and natural fibers are very attractive because they can be disposed of effortlessly after fulfilling their purpose without adversely affecting the environment [11,12].

Nevertheless, the practical use of bioplastics in various applications remains restricted due to their high costs, limitations of small-scale production, and occasional lower mechanical performance compared to petroleum-based plastics [13].

Cellulose is the most common organic polymer and is considered an almost inexhaustible source of raw material for responding to the increasing demand for environmentally friendly and biocompatible products. Cellulose is typically obtained from natural resources and agricultural waste, such as fibers, straw, or wood [14], and can be converted to different materials, such as bacterial, microcrystalline, or nanocrystalline cellulose products. Microcrystalline cellulose exhibits many useful characteristics and properties, including renewability, non-toxicity, biodegradability, high mechanical properties, large surface area, low density, and biocompatibility [15]. This renewable resource has been blended with biodegradable polymers, such as polylactic acid (PLA) [16,17], poly(butylene adipate-co terephthalate) (PBAT) [18,19], polycaprolactone (PCL) [20,21], or polyvinyl alcohol (PVA) [22]. The formulation of cellulose-biopolymer blends has specific applications in food packaging [23,24]. The current interest lies in the presence of cellulose in biopolymer matrices, which increases mechanical resistance, i.e., rigidity and the elastic modulus. The water barrier properties of the resulting blends are excellent because of the good interaction between the cellulose and biopolymer macromolecules. To improve the adhesion between cellulose particles and biopolymer matrices, a suitable compatibilizer, such as glycerol, can be introduced during the production of films by solvent casting. 

The most widely occurring biopolymer in nature after cellulose is chitin and it can be found in a range of eukaryotic species, such as crustacea, insects, and fungi [25]. Chitosan is the deacetylated form of chitin and is its most important derivative, and chitosan displays an excellent film-forming ability and exhibits good properties, including high transparency, antimicrobial activity, biocompatibility, biodegradability, and moderate water and oxygen permeability. Chitosan is insoluble in aqueous solutions, but it is soluble in diluted organic acid solutions due to the presence of amine groups, which are protonated in acid solutions. In order to prepare chitosan films, chitosan is usually dissolved in an acetic acid solution and the so-called solution casting technique is used to obtain the films [26]. Although chitosan is a very promising sustainable material, coming from natural sources, its current applicability in the packaging field is limited due to its poor mechanical performance and water barrier properties. 

However, chitosan–cellulose combinations are of particular interest. Because of their structural similarity, compatible blends that combine the physicochemical properties of chitosan with the excellent mechanical properties of cellulose can be formed [26,27,28,29,30]. To improve the compatibility between chitosan and cellulose, an adhesion promoter can be added to enhance the interfacial adhesion between the matrix and cellulose particles. An evolution of the effect of the presence of adipic acid in chitosan–cellulose combinations is carried out. The mechanical properties of physically cross-linked (uncured), chemically cross-linked (cured), and uncross-linked (prepared using acetic acid) films are compared. The presence of adipic acid improved the tensile strength of uncured and chemically cross-linked films more than 60% and 113%, respectively. Obviously, adipic acid is able to exert a compatibilizing effect, in addition to improving chitosan solubility. This greatly improves the overall mechanical behavior of these blends [26].

Interestingly, combinations of biopolymers, such as pectin and chitosan, and micro/nanocrystalline cellulose have been proposed also for innovative edible food packaging films [31]. The combination of chitosan, microcrystalline cellulose, and lignin for the formulation of films for active packaging has been reported [32]. Different combinations of the constituents and solvent casting conditions were exploited in an attempt to formulate films having improved mechanical behavior, UV barrier, and water vapor barrier. The presence of a microcrystalline cellulose setup to modify the absorption of UV irradiation, making the chitosan-based films more opaque, significantly reduces the water vapor permeability.

A biocompatible and nontoxic compound derived from food waste, mucic acid, is used as an alternative to acetic acid in chitosan solubilization and to cross-link the chitosan through ionic interactions and/or covalent amide bonds. This can promote the use of waste for the formulation of new sustainable materials that are biobased, biodegradable, and come from renewable sources, including wood and food waste. The biopolymer films were formulated by solvent casting, using renewable and sustainable materials, such as low-molecular-weight chitosan (Chit), microcrystalline cellulose (MCC), glycerol (Gly) as a plasticizer, and mucic acid (MA) as a substitute for the acetic acid (AA) and stabilizer. The formulated films were subjected to an FTIR and UV-visible spectroscopic analysis, mechanical test analysis, water contact angle, surface observation, and photo-oxidation evaluation. The occurrence of the degradation phenomena as a function of time was monitored using FTIR spectroscopy. The formulation of Chit-MCC-Gly-MA and Chit-MCC-Gly-AA films with similar performances and properties was demonstrated. The use of MA, rather than AA, during the preparation, represents the utilization of a sustainable additive for the improvement of the solubility of chitosan, and also has a beneficial effect on the photo-oxidation degradation of the film. 

## 2. Materials and Methods

### 2.1. Materials

Chitosan (Chit), with a low viscosity, deacetylation degree = 75–85%, and average molecular weight = 120 kg·mol^−1^, was purchased from Sigma Aldrich (St. Louis, MO, USA). Microcrystalline cellulose (MCC) was purchased from Alfa Aesar (Haverhill, MA, USA). Mucic acid (MA, also called galactaric acid; chemical formula: HOOC(CHOH)_4_COOH; molecular weight = 210.24 g·mol^−1^), acetic acid (AA, chemical formula: CH_3_CO_2_H; molecular weight: 60.05 g·mol^−1^), and glycerol (Gly, 1,2,3-Propanetriol; chemical formula: HOCH_2_CH(OH)CH_2_OH; molecular weight: 92.09 g·mol^−1^) were purchased from Sigma Aldrich and used without purification. Deionized water (DIW) was used in the entire study.

### 2.2. Preparation of Biocomposite Films

A solution casting technique was employed to form the composite film. Therefore, a solution of 25 mL was prepared with MA (0.28 g/25 mL) and stirred for 20 min at 100 °C to promote the formation of 1,4 lactone of the MA, which is more soluble in water; then, chitosan (0.45 g/25 mL) was added followed by stirring for 20 min at 50 °C until the chitosan was completely dissolved. An amount of MCC (1.125 g/25 mL) was added to the dissolved solution and stirred to disperse it evenly, and glycerin (0.4 g/25 mL) was added in order to improve the plasticity of the film. The polymer solution was kept under stirring overnight. The well-dispersed mixture was poured into a glass Petri dish at 50 °C to evaporate the water. The Chit-MCC-Gly-MA mass fraction was ca. 20/50/17.7/12.3 wt.%.

For comparison, other composite films were also prepared: Chit-AA, Chit-MA, Chit-MCC-Gly-AA, and Chit-MCC-Gly-MA. All the films created had thicknesses of about 100 μm.

### 2.3. Characterizations

#### 2.3.1. FT-IR Analysis

A Fourier Transform Infrared Spectrometer (Spectrum One, Perkin Elmer, Shelton, CT, USA) equipped with a Micro-ATR objective was used to record IR spectra using 16 scans at a resolution of 4 cm^−1^ in the attenuated total reflectance Fourier transform infrared (ATR-FTIR) mode in the range of 4000–500 cm^−1^, using air as the background. Measurements were taken for the chitosan, microcrystalline cellulose, and mucic acid powders. FTIR analysis was carried out to characterize the chemical structure by identifying the functional groups in the chitosan and Chit/MCC composite films, using 16 scans at a resolution of 4 cm^−1^. Measurements were obtained from the average of triplicate samples with a calculated maximum experimental error (relative standard deviation) of around 5%.

#### 2.3.2. UV–Visible Analysis

A UV–visible Spectrometer (Specord^®^250 Plus, Analytikjena, 24020 Torre Boldine (BG), Italy) was used to record the UV–Vis spectra performing eight scans between 200 and 900 nm at a resolution of 1 nm. The opacity values of the films are evaluated by using Equation (1):

(1) $$Opacity=\frac{{Abs}_{600}}{d}$$

where Abs600 is the value of absorbance at 600 nm and d is the film thickness (mm).

#### 2.3.3. Differential Scanning Calorimetry

A differential scanning calorimeter (Setaram, model DSC131 evo, Lyon, France) was used to investigate the calorimetric properties of the materials. The analysis was carried out with two cycles of heating from room temperature to 200 °C for the raw materials and to 250 °C for the composite films, at a 10 °C/min heating rate and one cooling cycle, with approximately the same weight (~5 mg) sealed in aluminum pans.

#### 2.3.4. Mechanical Characterization

Tensile tests were carried out using a Universal Testing Machine (Instron model 3365, Bucks, UK), equipped with a 1 kN load cell, following the ASTM D882 method, on rectangular samples (10 mm × 90 mm) cut by films prepared by solvent casting. The tests were performed using a tensile speed of 1 mm/min for 1 min in order to evaluate Young’s modulus, and then the velocity was increased to 10 mm/min until sample breakage and the elastic modulus (E) was calculated as the slope of the initial linear region of the stress–strain curves. The average values for elongation at break, EB; elastic modulus, E; and tensile strength, TS, were calculated.

#### 2.3.5. Morphological Analysis

The microstructure of the biocomposites was observed by using a Scanning Electron Microscope (Phenom ProX, Phenom-World, Eindhoven, The Netherlands) with an optical magnification range of 20–135x, electron magnification range of 80–130,000x, and acceleration voltage of 15 kV. The microscope was equipped with a temperature-controlled (25 °C) sample holder. The samples were positioned on an aluminum stub using an adhesive carbon tape. Prior to the SEM analysis, the samples were fractured in liquid nitrogen.

#### 2.3.6. Water Contact Angle Measurements

The water contact angle was measured using an OCA 20 (Data Physics Instruments, Filderstadt, Germany) apparatus equipped with a CCD camera and a high-performance digitizing adapter. The SCA 2.0 software (Data Physics Instruments) was used for data acquisition. The films were fixed on top of a plane solid support and kept flat during water deposition and acquisition. The sessile drop method was used with a droplet volume of 6 μL.

### 2.4. Photo-Oxidation Exposure

The photo-oxidation of composite films (about 100 µm thick) was carried out using a Q-UV-Solar Eye weatherometer (from Q-LAB, Westlake, OH, USA) equipped with UVB lamps (340 nm). The weathering conditions were continuous light exposure at T = 55 °C.

## 3. Results and Discussion

### 3.1. Spectroscopy and Contact Angle Analysis

**Figure 1:**
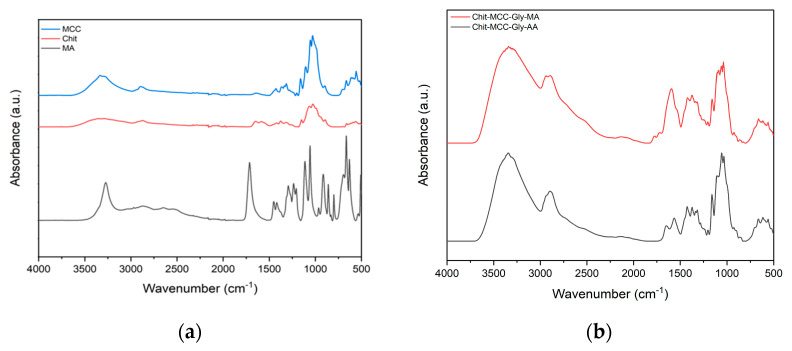
(**a**) ATR of microcrystalline cellulose (MCC), chitosan (Chit), and mucic acid (MA); (**b**) FTIR of composite films with mucic acid (red line) and acetic acid (black line).

To identify the main functional groups of raw materials, chitosan, microcrystalline cellulose, and mucic acid, we plotted the ATR-FTIR spectrum in Figure 1a. Furthermore, to investigate the structural changes in the Chit/MCC-based samples, an FTIR analysis was carried out, and the obtained FTIR spectra are plotted in Figure 1b. As is noticeable in Figure 1a, chitosan and microcrystalline cellulose are biopolymers with similar structures containing various functional groups, such as hydroxyl (-OH), amine (-NH_2_), and carbonyl (C=O) groups. 

The absorbance peaks located in the ranges of 3600–3100 cm^−1^ and 2900–2800 cm^−1^ are attributed to the stretching of OH groups and aliphatic-saturated CH, respectively [33]. The peak at about 1641 cm^−1^ is associated with both the H-O-H bending of absorbed water molecules of the microcrystalline cellulose with the intensity dependent on the residual hemicellulose content in the MCC [24,34,35], and the C=O stretching of the secondary amide group of chitosan [36]. Therefore, the spectrum of mucic acid shows one absorbance signal at about 1725 cm^−1^, which is related to carboxylic acid carbonyls [37].

Figure 1b shows the FTIR spectra of the composite films containing chitosan, microcrystalline cellulose and glycerol (Chit-MCC-Gly), and mucic acid (MA; red line) or acetic acid (AA; black line). The glycerol’s presence is detectable in the range of 1125–1100 cm^−1^, and this peak can be attributed to the vibration of the CH-OH bond while the stretching of the O-H bond occurs in the range of 1400–1200 cm^−1^. A visual examination of the spectra suggests that the presence of MA can be observed in the peaks in the range of 1650–1800 cm^−1^ and this has no significant influence on the appearance of the other peaks. The broad absorption band, which appears in the range of 3700–2800 cm^−1^, is related to the collective absorption by both O-H and N-H groups in the biopolymer, and this is an indication of the bonding of chitosan with MCC through hydrogen bonds. In the spectra of both Chit-MCC-Gly-MA and Chit-MCC-Gly-AA, the complex band in the range of 3700–2800 cm^−1^, due to the stretching vibrations of O-H and N-H, was shifted to higher wave numbers, in comparison to the neat Chit sample, suggesting that strong interactions occurred between the MCC and -NH_2_ groups of chitosan. The existence of hydrogen bonding indicated that Chit and MCC had relatively good compatibility, and this further improved the performance of the composite materials [38,39].

**Figure 2:**
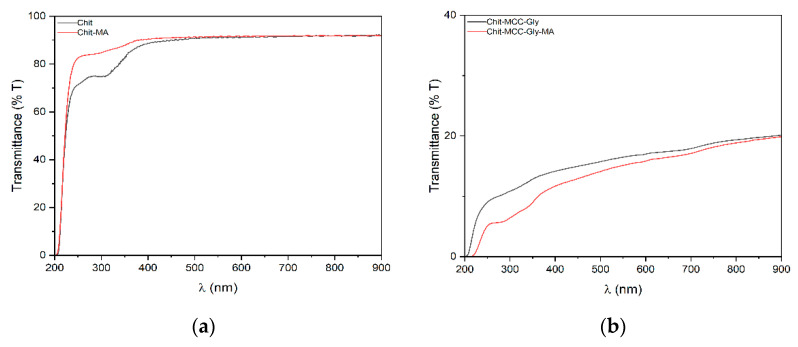
UV-vis spectra of (**a**) Chit composite films and (**b**) Chit/MCC composite films.

In Figure 2a,b, the UV–visible spectra of Chit- and Chit/MCC-based composite films are plotted. Opacity values were calculated, considering the absorbance values at 600 nm and the thicknesses of the samples, and using Equation (1), reported in the experimental section, Section 2.3.2; all obtained values are shown in Table 1. As expected, the presence of MCC leads to a significant increase in film opacity, and this is very important to take into consideration when designing the applications for these biocomposite films.

The UV–vis spectra of the films show a broad peak between 270 and 350 nm, and according to the literature [36], this peak can be attributed to the oxidation of the thin film during solution casting. Therefore, the Chit-MA film appears more transparent than the Chit-MCC-Gly-MA film because of the MCC’s white color. Consequently, it can be assumed that the composite film provides greater protection against UV rays.

Therefore, to evaluate the influence of mucic acid presence on film wettability, contact angle measurements were performed, and the obtained results are shown in Table 1. The values of the water droplet angle, just after water droplet deposition, increased due to MA presence, and even more due to MCC presence, suggesting the formation of a more hydrophobic surface. As is noticeable, the MCC’s presence has a beneficial effect on the hydrophobic properties, making the chitosan-based film less likely to absorb into coordinate water molecules. Interestingly, also the MA’s presence has a beneficial effect on the hydrophobic properties, contributing to the formation of chitosan-based films with improved hydrophobicity. 

### 3.2. Thermal Properties

**Figure 3:**
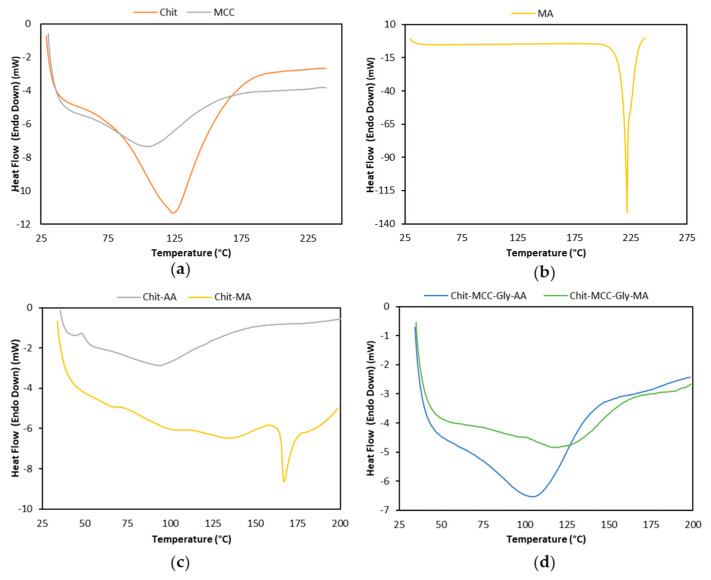
Differential scanning calorimetry (DSC) thermograms recorded during the first heating scan of raw materials and composite film: (**a**) Chit and MCC powders; (**b**) MA powder; (**c**) Chit-based films; and (**d**) Chit/MCC-based biocomposite films.

As for the DSC analysis, the first heating cycle contained relevant information regarding both thermal history and structural properties. Figure 3 shows the thermograms of the first heating scan and Table 2 summarizes the main thermal properties, i.e., temperature of on-set melting (T_on_), melting temperature (T_m_), and fusion enthalpy (ΔH) of the raw materials and Chit/MCC-based biocomposite films.

The thermal events in the range of 40–250 °C, related to significant changes in the thermal behavior, are noticeable for all investigated materials (see Figure 3a–d). Neat Chit and MCC show melting temperatures of 124.1 °C and 105.24 °C, respectively, and reasonably high values of fusion enthalpies: 357.64 and 205.14 J g^−1^, respectively. It is worth noting that neat MA shows a very high melting temperature at 224.2 °C, and a significant high value of fusion enthalpy at 560.6 J g^−1^, suggesting that it is a thermally stable and very crystalline material. 

As is noticeable in Figure 3c, the addition of MA to Chit leads to the occurrence of complex thermal events that begin at ca. 73.5 °C and show three melting peaks at 101.7 °C, 136.9 °C, and 167.0 °C, probably due to the formation of Chit–MA complex crystalline aggregate structures. However, the presence of MA in the Chit film significantly increases both the melting temperature and fusion enthalpy, in comparison to the values for the Chit-AA film, making the Chit-MA film more thermally stable.

The Chit-MCC-Gly biocomposite film is more thermally stable, in comparison to neat Chit films, due to the presence of both MCC and Gly, and this is even more pronounced in the film produced with MA. The trends, shown in Figure 3d, highlight that the presence of MA in the Chit/MCC/Gly biocomposite films causes a shift in the average event temperature to 120.0 °C from the 106.4 °C peak detected for the biocomposite films produced with AA. Furthermore, MCC and Gly exert contrasting effects on the thermal behavior; specifically, MCC exerts a nucleating effect, while the Gly has a plasticizing effect, decreasing the system’s crystallinity. 

### 3.3. Mechanical and Morphological Observations

**Figure 4:**
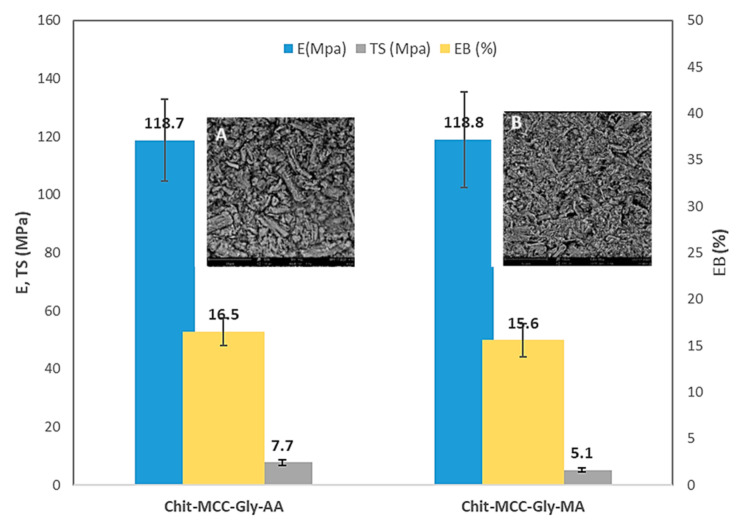
Average values of main mechanical properties: elastic modulus (blue) (MPa), tensile strength (gray) (MPa), and elongation at break (yellow) [%], of investigated biocomposite films. Inserts show the surfaces of (**A**) Chit-MCC-Gly-AA and (**B**) Chit-MCC-Gly-MA.

To evaluate the mechanical behavior, the films were subjected to a tensile test, and the obtained results of the elastic modulus (E), tensile strength (TS), and elongation at break (EB) are plotted in Figure 4. 

Gly was added to the Chit/MCC biocomposite films to improve the interaction between Chit and MCC; this was because the glycerol molecules were able to penetrate through the biopolymer chains, interfering with them. Furthermore, according to the literature [32], glycerol molecules decrease the intermolecular attractions, increasing the biopolymer chain’s mobility. The results show no significant differences between the films produced with MA and AA, suggesting that MA can be considered a good candidate for the preparation of Chit/MCC-based films.

However, as is noticeable in the inserts in Figure 4, the surfaces of both Chit-MCC-Gly-AA (see insert A) and Chit-MCC-Gly-MA (see insert B) biocomposite films appear very similar. As is noticeable, both Chit-MCC-Gly-AA and Chit-MCC-Gly-MA show randomly oriented microcrystalline particles that are well dispersed into the chitosan matrix, and it is not possible to observe any substantial differences. The latter outcome again suggests that the MA can be considered a valid candidate for the preparation of Chit/MCC-based films.

### 3.4. Photo-Oxidation Resistance

**Figure 5:**
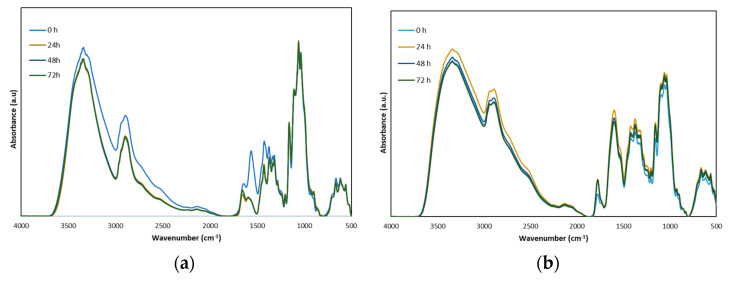
FTIR spectra of (**a**) Chit-MCC-Gly-AA biocomposite films and (**b**) Chit-MCC-Gly-MA biocomposite films, as a function of exposure time.

To simulate the degradation phenomena that occur in in-service conditions, the Chit-MCC-Gly-AA and Chit-MCC-Gly-MA biocomposite films were subjected to accelerated photo-oxidation using UVB lamps (313 nm). The progress of photo-oxidative degradation over time was monitored by FTIR analysis and, in Figure 5a,b, the obtained spectra at different time intervals are plotted.

However, according to the literature [36,40,41,42], chitosan degradation occurs mainly by depolymerization, followed by deacetylation; oxidation, if the oxygen is available; and random interchain cross-linking. Overall, the photo-oxidation of chitosan-based materials can be profitable following the monitoring of the changes in the complex peak in the range of 2000–1500 cm^−1^, which can be assigned to the formation of oxygen-containing products due to the degradation over time. 

Although the FTIR spectra of Chit-MCC-Gly-AA and Chit-MCC-Gly-MA before the photo-oxidation (0 h), shown in Figure 1b, are very similar, a slight difference in the range of 2000–1500 cm^−1^ is noticeable because of the presence of MA containing intrinsic carboxylic groups. Interestingly, the peaks in the range of 2000–1500 cm^−1^ slightly decreased for both Chit-MCC-Gly-AA and Chit-MCC-Gly-MA after 24 h of exposure, probably because of the loss of coordinated water molecules during the first stage of UVB exposure. It is worth noting that this decrease in the complex peak in the range of 2000–1500 cm^−1^ is more pronounced for the Chit-MCC-Gly-AA biocomposite film, rather than for Chit-MCC-Gly-MA. Furthermore, the Chit-MCC-Gly-AA spectra show a gradual increase in the small peak at ca. 1715 cm^−1^, and, probably, this can be attributed to the formation of new oxygen-containing products, suggesting lower photo-oxidation resistance in comparison to the resistance of the Chit-MCC-Gly-MA biocomposite film. The latter suggests that the presence of MA has a beneficial effect on the protection of Chit-MCC-Gly biocomposite films against photo-oxidation during UVB exposure.

However, probably, the good performance of MA against UVB exposure can be understood considering that the MA improves chitosan’s solubility through its protonation, and hydrogen donation can also have a radical saturation effect. According to the literature, MA can act as an antioxidant, and some natural antioxidants can also exert protection when scavenging some radicals [41,42]. As is well-known, AA can protonate the chitosan, but it is not able to act as an antioxidant. 

## 4. Conclusions

Biocomposite films based on naturally occurring materials, such as chitosan, microcrystalline cellulose, and mucic acid, were successfully formulated by solvent casting and subjected to analyses by spectroscopy, tensile test, water contact angle measurements and surface observations, differential scanning calorimetry, and accelerated UVB exposure to evaluate their photo-oxidation resistance. Both Chit-MCC-Gly-AA and Chit-MCC-Gly-MA showed very similar properties and performances, although the replacement of AA by MA was advantageous in terms of the thermal behavior and photo-oxidation resistance of the films. It is worth noting that MA can successfully replace classical AA in the formulation of chitosan and chitosan/microcrystalline cellulose-based films, and the formulated films show slightly improved thermal properties and photo-oxidation resistance. Therefore, based on all the obtained results, it is possible to conclude that the formulated Chit-MCC-Gly-MA and Chit-MCC-Gly-AA films show similar performances and properties, and the advantage of using MA, rather than AA, consists of the identification of sustainable additives to improve the solubility of chitosan, having also a beneficial effect on the photo-oxidation degradation property.

Therefore, the importance of this work lies in in the possibility of formulating films based on naturally occurring and waste materials, with good properties and oxidation resistance, following the principles of achieving a more sustainable outcome and considering natural and waste feedstocks for biocomposite film formulations.

## Figures and Tables

**Table 1:** Calculated values of opacity and measured water contact angles (θi) of the investigated samples.

Sample	Opacity (A mm^−1^)	WCA (°)
Chit-AA	0.39	80.1 ± 2.5
Chit-MA	0.38	82.5 ± 2.7
Chit-MCC-Gly-AA	7.69	91.1 ± 3.0
Chit-MCC-Gly-MA	8.89	93.9 ± 2.3

**Table 2:** Average values of main thermal properties of raw materials and composite films.

Sample	T_on_ (°C)	T_m_ (°C)	ΔH (J g^−1^)
Chit powder	73.5	124.1	357.64
MCC powder	58.4	105.2	205.14
MA powder	219.7	224.2	560.6
Chit-AA film	48.72	94.1	161.24
Chit-MA film	73.5	101.7; 136.9; 167.0	178.33
Chit-MCC-Gly-AA film	74.4	106.4	147.21
Chit-MCC-Gly-MA film	75.1	120.0	114.54

## Data Availability

Data are contained within the article.
